# Challenges in the Management of Severe Serotonin Syndrome: A Case Report

**DOI:** 10.7759/cureus.76012

**Published:** 2024-12-19

**Authors:** Pegah Yakhchalian, Kimia Mondegari, Rahi Reza Daneshvar

**Affiliations:** 1 Psychiatry and Behavioral Sciences, Kaweah Delta Health Care District, Visalia, USA

**Keywords:** bupropion and serotonin syndrome, bupropion overdose, medication overdose, serotonin syndrome and autonomic instability, serotonin syndrome management

## Abstract

Serotonin syndrome (SS) is a potentially life-threatening condition that can result from excessive serotonergic activity, often due to SSRIs, formally known as selective serotonin reuptake inhibitors. The syndrome and its effects are often secondary to drug-drug interactions or dose-related. This case highlights a 47-year-old female who presents with a history of major depressive disorder, generalized anxiety disorder, and severe alcohol use disorder. She was brought to the Emergency Department after attempting suicide by ingesting an inordinate amount of bupropion (Wellbutrin). Although bupropion is not a direct serotonergic reuptake inhibitor, its inhibition of the CYP2D6 pathway has the potential to elevate the levels of alternative serotonergic agents, potentially leading to SS. The patient’s symptoms were inclusive of autonomic instability and clonus, which are consistent with this syndrome, and which necessitated the discontinuation of all serotonergic medications, in addition to the initiation of supportive care. Management included benzodiazepines, guanfacine, and gabapentin to mitigate the glutamatergic activity SS can induce. This case emphasizes the need to closely monitor those prescribed this class of medications, while concomitantly incorporating interdisciplinary collaborative management for those afflicted with SS.

## Introduction

Serotonin syndrome (SS) is a life-threatening adverse medication reaction resulting from excessive serotonergic activity, often due to drug-to-drug interactions, poisoning, or, less commonly, the expected therapeutic effect a single drug can potentially elicit [1]. Though this patient ingested a large amount of bupropion (Wellbutrin), which inhibits norepinephrine and dopamine, it is not known to have direct serotonin agonist properties nor inhibit serotonin reuptake activity, elevating serotonin’s availability. Nevertheless, it has still been linked to cases of individuals affected by SS. This association likely arises from bupropion's established inhibition of the CYP2D6 enzyme, which can lead to elevated blood levels of selective serotonin reuptake inhibitors (SSRIs), tricyclic antidepressants, and other medications, such as buspirone, opioids, ondansetron, and hydralazine [2].

A recent review by the Toxicology Investigator's Consortium (ToxIC) found that medical toxicologists often diagnose SS in approximately 6% of single-agent bupropion overdoses [3]. Diagnosing SS can be challenging due to its nonspecific clinical presentation and the lack of confirmatory laboratory tests. A high level of suspicion, combined with a thorough history, physical examination, knowledge of exposure to serotonergic agents, and use of the Hunter Criteria, aids in diagnosing SS [4].

Bupropion is a monocyclic phenylethylamine antidepressant and is the only Food and Drug Administration (FDA)-approved synthetic cathinone indicated as monotherapy or as adjunctive treatment for major depressive disorder, seasonal affective disorder, and smoking cessation. Between 2000 and 2013, reports of bupropion exposures to the National Poison Data System (NPDS) increased by 75%. While bupropion is effective, seizures are known to be a potentially significant adverse effect. In addition to seizure activity, an overdose of bupropion is also associated with tachycardia and agitation; however, it may also be an atypical finding when an individual is experiencing serotonin toxicity [3].

The following will present a case of a middle-aged woman who attempted suicide by ingesting 70 tablets of 150 mg extended-release (XL) bupropion. Following this episode, she experienced multiple medical insults, including a seizure disorder, which necessitated intubation and the placement of a percutaneous endoscopic gastrostomy (PEG) tube for respiratory and nutritional support. We will next see how the case evolved and how it was subsequently managed by different medical teams who collaborated and decided upon specific interventions to save her life.

## Case presentation

This is a case of a 47-year-old female who is employed at an insurance company and has a known psychiatric history of major depression, generalized anxiety disorder, and severe alcohol use disorder. Her alcohol consumption has been significant, as she stated that she has been consuming approximately one liter of vodka daily, four times weekly. As a result, approximately nine months ago, she was initiated on Buspar 30 mg twice daily, bupropion 450 mg daily, quetiapine 200 mg nightly, and Xanax 0.5 mg PRN for anxiety.

She unfortunately arrived at the Emergency Department following a suicide attempt, as reported by her husband, who noted she had ingested approximately 70 tablets of bupropion XL 150 mg after they had an argument. In addition, she inflicted a 5 cm laceration on her left wrist that required suturing for closure. Upon initial assessment in the Emergency Department, the patient was alert and oriented, but about two hours later, after arrival, she began experiencing seizure activity, resulting in the need for intubation to protect her airway. She subsequently developed QT prolongation (620-660 ms), which led to cardiac arrest the next day. This necessitated cardiopulmonary resuscitation (CPR) to be initiated, which ultimately resulted in her successfully being resuscitated. She was subsequently initiated on dexmedetomidine at 0.5 mcg/kg/hr for continuous sedation, but during attempts to wean her from this medication, she became increasingly agitated.

Six days later, while attempting to extubate her, she developed significant tachypnea in addition to difficulty managing excessive mucoid secretions, necessitating reintubation. Following another attempt to extubate her and taper the dexmedetomidine, she had a tracheostomy and a PEG tube placed for continuous respiratory and nutritional support. She was subsequently transferred to the Intermediate Critical Care Unit (ICCU) for management. After a psychiatry consult was initiated, the liaison team found her to exhibit inducible clonus in both hands while also presenting with a high fever noted to be 39°C. The persistence of autonomic instability remained evident, as in addition to the fever and clonus, she also exhibited episodes of severe diarrhea, all of which are signs of SS. They based their conclusions on Hunter’s Criteria and subsequently adjusted her psychotropic medications. As a result, all serotonergic agents, including opioids, were discontinued. Unfortunately, after the patient was admitted to the ICCU from the Emergency Department, the medical team resumed all her home medications (including bupropion) and even added opioids and hydralazine to her inpatient treatment plan. The combination of these medications likely played a role in inducing her decompensation, as their cumulative effects potentially heightened the development and worsening of symptoms of SS. The psychiatry liaison team again recommended the discontinuation of all psychotropic medications and began treating her as required. Through appropriate management, she was ultimately stabilized and transferred to a mental health facility for further psychiatric assessment and treatment.

## Discussion

SS occurs due to excessive stimulation of serotonin receptors in the central and peripheral nervous systems, typically triggered by medications that elevate synaptic serotonin levels. Serotonin binds to various families of 5-HT receptors, though there is no single receptor known to be solely responsible for SS. The literature widely acknowledges that the 5-HT1A and 5-HT2A subtypes (particularly 5-HT2A) play significant roles in SS’s manifestations. Second-generation antipsychotics are also associated with SS due to their effects on dopamine (D2) and serotonin receptors, particularly through antagonism of the 5-HT2A receptors and partial agonist activity at the 5-HT1A receptors. Although the precise mechanism remains unclear, it is hypothesized that 5-HT2A receptor antagonism may indirectly increase serotonin levels, leading to subsequent activation of 5-HT1A receptors, which can potentially contribute to SS [5].

Although bupropion is not known to directly stimulate serotonin receptors nor interfere with serotonin reuptake, it has been identified as a potential contributor to SS. Cases of SS have indeed been reported in connection with bupropion; however, primarily, they were cases of overdose or when bupropion was co-administered with other drugs known to have serotonergic effects. The potential involvement of bupropion in SS likely arises from its established inhibition of the cytochrome P4502D6 pathway. This inhibition can lead to elevated blood levels of serotonin. In addition, SSRIs, tricyclic antidepressants, and medications such as buspirone, hydralazine, and opioids can all potentially cause this identical effect [6]. An attempt was made to reduce her sedation by tapering her from dexmedetomidine. However, as she had already been under its influence for more than 24 hours, it led to withdrawal symptoms, which resulted in severe agitation in addition to causing her vital signs to become unstable [7]. Therefore, cross-titration with guanfacine was suggested as an alternative due to fewer cardiovascular side effects. We selected guanfacine over clonidine, which was also an effective option but could lead to hypotension. Guanfacine and clonidine differ in their effects on hypertension management. Guanfacine has significantly higher selectivity for presynaptic alpha-adrenoceptors and a much greater alpha 2/alpha 1 selectivity ratio than clonidine. While clonidine decreases cardiac output, guanfacine lowers peripheral resistance and increases stroke volume. Additionally, guanfacine withdrawal symptoms are milder and delayed compared to clonidine. Overall, guanfacine may be preferable as a central alpha-adrenoceptor stimulant for treating hypertension [8].

Benzodiazepines were ultimately initiated as the mainstay of treatment [9], while gabapentin was also added to offer presynaptic inhibitory effects for glutamatergic neurons. In experimental settings, gabapentin decreased the amplitude of excitatory postsynaptic currents in certain neurons, likely due to the reduced release of excitatory amino acids from presynaptic terminals. These effects support its potential for managing symptoms related to excessive glutamate activity, including certain types of neuropathic pain [10]. Animal studies have shed light on the pathophysiology of SS, revealing that 5-HT2A receptor stimulation causes hyperthermia, while 5-HT1A receptor stimulation leads to hypothermia. The hyperthermia mechanism likely involves increased norepinephrine, dopamine, and glutamate in the hypothalamus and frontal cortex. Certain medications, such as the N-methyl-D-aspartate (NMDA) antagonist memantine and gamma-aminobutyric acid type A (GABAA) receptor modulators, effectively reduce hyperthermia in rat models experiencing SS, indicating a potential role for GABA and NMDA receptors in the condition's pathogenesis [11]. As the patient exhibited autonomic instability, hydralazine should have been avoided due to its significant inhibitory effects on monoamine oxidase (MAO), which could exacerbate serotonin toxicity. Instead, the patient would likely have experienced an improved beneficial effect if she had been initiated on rapidly acting medications with known short half-lives, such as esmolol, to mitigate the hypotensive risk [1]. Cyproheptadine was considered but not initiated, as the patient showed significant improvement within 24 hours with the medication management as mentioned above.

## Conclusions

Optimal management of SS hinges on two vital components: supportive care and meticulous risk assessment, coupled with vigilant monitoring of those demonstrating mild to moderate SS to prevent its progression to life-threatening severity. This endeavor demands interdisciplinary collaboration between the psychiatric liaison and medical teams. Proactive consultation with the psychiatry consult liaison team upon arrival could have prevented the reinstatement of home medications, thereby mitigating the risk of SS. In addition, it’s vital to select the correct medications and dosages when considering a patient’s treatment plan in the presence of SS. As it relates to the patient in this case study, the continuation of opioids, hydralazine, ondansetron, the initial low dose of benzodiazepine, and the continuation of dexmedetomidine may not align with optimal treatment strategies.
